# Cellular senescence in the aging and diseased kidney

**DOI:** 10.1007/s12079-017-0434-2

**Published:** 2017-12-19

**Authors:** F. A. Valentijn, L. L. Falke, T. Q. Nguyen, Roel Goldschmeding

**Affiliations:** 1 Department of Pathology, University Medical Center Utrecht, H04.312, Heidelberglaan 110, 3584 CX Utrecht, The Netherlands; 2 Department of Internal Medicine, Diakonessenhuis, Utrecht, The Netherlands

**Keywords:** Apoptotic balance, DNA damage response, Cellular senescence, Renal aging, Renal disease, Senotherapy

## Abstract

The program of cellular senescence is involved in both the G1 and G2 phase of the cell cycle, limiting G1/S and G2/M progression respectively, and resulting in prolonged cell cycle arrest. Cellular senescence is involved in normal wound healing. However, multiple organs display increased senescent cell numbers both during natural aging and after injury, suggesting that senescent cells can have beneficial as well as detrimental effects in organismal aging and disease. Also in the kidney, senescent cells accumulate in various compartments with advancing age and renal disease. In experimental studies, forced apoptosis induction through the clearance of senescent cells leads to better preservation of kidney function during aging. Recent groundbreaking studies demonstrate that senescent cell depletion through INK-ATTAC transgene-mediated or cell-penetrating FOXO4-DRI peptide induced forced apoptosis, reduced age-associated damage and dysfunction in multiple organs, in particular the kidney, and increased performance and lifespan. Senescence is also involved in oncology and therapeutic depletion of senescent cells by senolytic drugs has been studied in experimental and human cancers. Although studies with senolytic drugs in models of kidney injury are lacking, their dose limiting side effects on other organs suggest that targeted delivery might be needed for successful application of senolytic drugs for treatment of kidney disease. In this review, we discuss (i) current understanding of the mechanisms and associated pathways of senescence, (ii) evidence of senescence occurrence and causality with organ injury, and (iii) therapeutic strategies for senescence depletion (senotherapy) including targeting, all in the context of renal aging and disease.

## Renal aging and disease

The pathophysiological substrate of chronic kidney disease (CKD) is kidney injury leading to fibrosis and reduced kidney function reflected in a lower glomerular filtration rate (GFR). This might be due to the normal wear and tear associated with aging, and/or to renal disease. In the developed world, life expectancy has increased substantially and this is accompanied by a growing portion of the population, particularly elderly, diagnosed with CKD (Coresh et al. 2007). In 2016, the global prevalence of CKD was estimated to be as much as 13.4%, forming a global health burden with a high economic cost to health systems worldwide (Hill et al. 2016). CKD patients are highly susceptible to additional injury, subsequent development of end stage renal disease (ESRD) and ultimately death, all of which are thought to be caused by lack of adequate repair. The mechanisms leading to fibrosis in both renal aging and renal damage are complex and involve multiple pathological phenomena and signaling pathways, such as pro-inflammatory/fibrotic signaling, loss of renoprotective factors (e.g. Klotho and bone morphogenetic proteins), vascular rarefaction and oxidative stress (O'Sullivan et al. 2017). A relatively new theory suggests involvement of cellular senescence as a central process in both early and late phases of renal aging and injury, connecting existing mechanisms of fibrosis.

## Cellular senescence and associated pathways

**Fig. 1:**
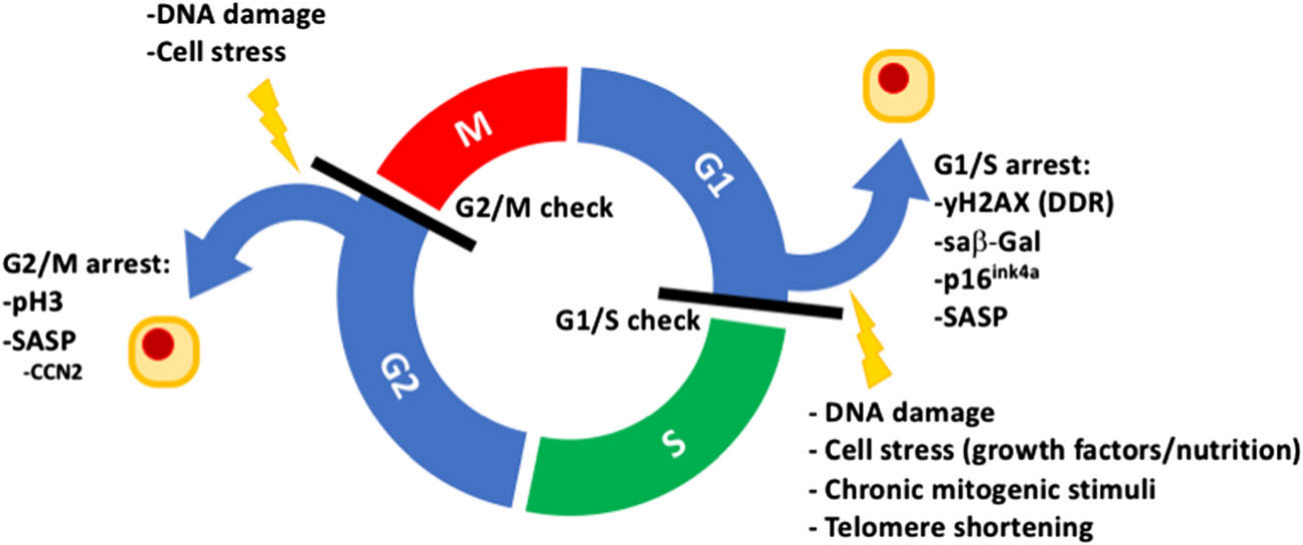
The cell cycle, relevant cell cycle arrest points, and their inducers and associated markers

Cellular senescence traditionally refers to a permanent cell cycle arrest (CCA) that can be initiated by various cellular stresses despite the presence of growth-inducing stimuli. This is opposed to quiescence, which is a temporary state of CCA due to a lack of growth stimulation (Blagosklonny 2011). Senescence has typically been linked to the G1-phase of the cell-cycle, but it does occur also in G2 (see below) (Stein and Dulić 1995; Smith and Pereira-Smith 1996). Major triggers of senescence include repeated cell division and telomere shortening (also referred to as replicative senescence), and factors such as oxidative stress or genotoxic injury (stress–induced premature senescence) (Fig. 1) (Campisi 1997; Toussaint et al. 2000). Additionally, a number of cytokines/growth factors have been implicated to induce senescense, including transforming growth factor-β (TGF-β) and the matricellular protein CCN1 (Datto et al. 1995; Jun and Lau 2010; Kim et al. 2013). Mechanistically, senescent cells can act cell-autonomously by induction of CCA and non-cell-autonomously by influencing neighboring cells through proteins that are part of the senescence-associated secretory phenotype (SASP) (Xue et al. 2007).

## Senescence-associated secretory phenotype

Senescent cells have a distinct secretome termed the senescence-associated secretory phenotype (SASP)(Krtolica et al. 2001; Coppé et al. 2008). Via this SASP, senescent cells affect neighboring cells by producing pro-fibrotic and pro-inflammatory factors, including interleukin 6, plasminogen activator inhibitor-1 (PAI-1), TGF-β and connective tissue growth factor (CCN2/CTGF), several of which are considered markers of senescence (Table 1)(Matjusaitis et al. 2016a). Normally, this immunogenic phenotype is part of a cancer defense mechanism that enables senescent cells to be eliminated (senescent-cell clearance) by the immune system through a process known as immune surveillance (Kang et al. 2011). However, an impaired immune system due to aging, disease or immunosuppressive therapy may cause senescent cells to evade elimination and maintain their metabolic activity. Although the exact composition of the SASP secrotome is unknown, it is considered to be a driving force behind senescence-induced fibrosis of the kidney (Sturmlechner et al. 2017).

**Table 1:** Features of senescent cells

Feature	Senescence marker	Method of detection
Senescence-associated secretory phenotype	Cytokines (IL-6, IL-8, GROα, GROβ, IL- α, PAI-1, CCL2/MCP-1) Growth factors (GM-CSF, G-CSF, HGF/SF, IGF, TGF-β, CCN2/CTGF) Proteases (MMP-1, −2, and −3) Non-soluble extracellular matrix proteins (collagens, fibronectin, laminin)	ELISA, FACS
DNA-associated	DNA damage markers (γH2AX, ATM, ATR, TP53, Rad17, MDC1, TIF) DNA synthesis (Ki67, EdU, BrdU) Telomere length/dysfunction Epigenitic changes (senescence-associated heterochomatin foci)	IHC Ki67 IHC; EdU or BrdU incorporation qPCR, FISH CiA, DAPI staining, IHC
DNA-damage response	Proteasome activity Lysosomal activity (β-galactosidase, α-Fucosidase) ROS	Fluorogenic peptide substrate assay IHC, qPCR, EM, WB DCFDH-DA fluorometry, chemiluminescent oxygen detection reagents, FACS
Cell cycle arrest	Cyclin-dependent kinase inhibitors (p21CIP1, p16INKa, p19ARF, p14ARF, p27KIP1, p15Ink4b)	IHC, qPCR

Abbreviations: IHC = immunohistochemistry; qPCR = quantitative PCR; EM = electron microscopy; WB = Western blot

Senescent cells express a broad spectrum of features, rendering a distinct morphology in vivo (reviewed by ref. (Sharpless and Sherr 2015)). Typical phenotypic traits are permanent CCA, persistent DDR, SASP, epigenetic changes like senescence-associated heterochromatic foci (SAHFs), altered metabolism including increased lysosomal and proteasomal activity, and telomere shortening and dysfunction (Sharpless and Sherr 2015; Matjusaitis et al. 2016b). The most commonly used biomarkers of senescence are senescence-associated β-galactosidase (SA-β-Gal) and p16. *(,,*(Dimri et al. 1995; Krishnamurthy et al. 2004; Burd et al. 2013)*).* Despite the availability of several markers and detection techniques (e.g. immunohistochemistry), accurate detection of senescent cells is complicated by (i) heterogeneity of senescent cells, (ii) organismal and possibly even individual variation of senescent markers and (iii) low sensitivity and specificity of senescent markers. (Gil and Peters 2006; Aan et al. 2013). Therefore, it is important to use combinations of different markers to reliably identify senescent cells.

## Prolonged cell-cycle arrest

Prolonged CCA is a key feature of senescence and is mediated via induction of the DDR. Following DNA damage, the DDR arrests cell cycle progression at specific checkpoints, particularly the G1/S checkpoint, thereby allowing time for DNA repair to prevent that errors are replicated or passed on to daughter cells in mitosis (Jackson and Bartek 2009). Cells with repairable DNA lesions go into transient CCA (quiescence), eventually re-entering the cell cycle in case of adequate DNA damage response by the DDR machinery. In contrast, severe or irreparable DNA lesions trigger prolonged DDR signaling, resulting in apoptosis or permanent growth arrest (senescence) (Campisi and d'Adda di Fagagna 2007).

**Fig. 2:**
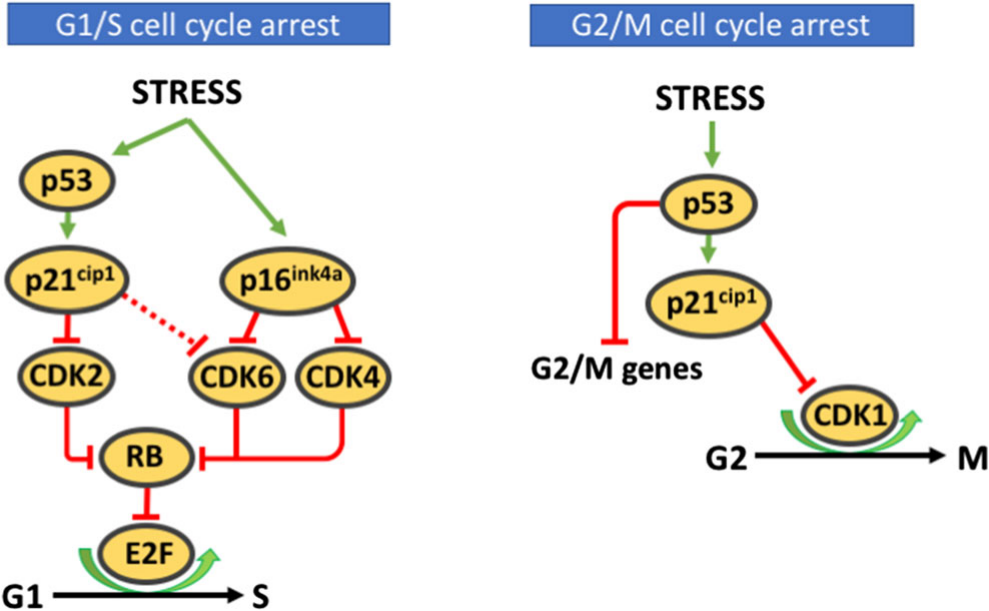
Cell cycle arrest signaling. Left panel: Major signaling pathway associated with G1S arrest. Right panel: Major signaling pathway associated with G2 M arrest

Senescence is classically linked to the G1-phase of the cell-cycle (Stein and Dulić 1995; Smith and Pereira-Smith 1996). *(,)* However, accumulating evidence indicates that senescence also occurs in the G2 phase, generally referred to as G2-arrest. (reviewed in ref. (Gire and Dulic 2015)*.* It is widely accepted that senescence associated prolonged G1- and G2-arrest occurs via late anti-proliferative DDR signaling in response to persistent DNA damage (Malaquin et al. 2015). Cell cycle progression requires activation of cyclin dependent kinases (CDKs). DDR induced prolonged CCA in senescence is characterized by accumulation of cyclin dependent kinase inhibitors (CKIs) like tumor protein p53 (TP53 or p53), p21^CIP1^ (p21) and p16^INK4a^ (p16) (el-Deiry et al. 1993; Harper et al. 1993). These CKIs inactivate CDKs and block CDK-mediated phosphorylation of the retinoblastoma tumor suppressor (Rb). This causes Rb to remain attached to and thereby inhibit the transcriptionally active E2F protein complex, thus preventing G1/S transition and DNA replication, or G2/M progression and mitosis, ultimately limiting cellular proliferation (Zhang et al. 1993; Serrano et al. 1993; Jullien et al. 2013) (Fig. 2).

Several relevant differences between G1- and G2-arrest are postulated. Firstly, replicative senescence applies mainly to G2 arrest as telomere attrition preferentially triggers DDR at the G2/M checkpoint (d'Adda di Fagagna 2008; Jullien et al. 2013; Mao et al. 2014). Secondly, p53 mediates senescence independent of p21 in the G2 phase (Johmura et al. 2014). Thirdly, the G2/M checkpoint is not as efficient in inducing CCA as the G1-S checkpoint, which relies on strong p21 induction (Löbrich and Jeggo 2007; Cesare et al. 2013). Prolonged (i.e. senescent) G2-arrested cells express enhanced levels of profibrotic growth factors like TGF-β1 and CCN2 (Yang et al. 2010). Hence, the term “maladaptive regeneration/repair” has been postulated (Bonventre 2014a). Yet, to our knowledge the only specific marker for G2-arrest to date is histone H3 phosphorylation at Ser10 (p-H3), as cells in G2 phase show a distinct phosphorylation and IHC staining pattern of p-H3. Other markers to make a clear distinction between G1- and G2-arrested cells are lacking, and also the possible variability of the exact composition of the SASP, is still poorly defined, thus it seems appropriate to refer to cellular senescence-like features, rather than suggesting that these identify a homogeneous phenotype (Crosio et al. 2002; Yang et al. 2010).

## The apoptotic balance

The p53 protein plays an important role in controlling cell fate by mediating temporary CCA, senescence, or apoptosis in case of irreversible DNA damage. The absolute levels of p53 expression seem to be decisive in determining cell fate (Khoo et al. 2014). High levels of p53 lead to apoptosis and lower levels result in temporary CCA (Chen et al. 1996; Kracikova et al. 2013). However, the effect of p53 on senescence remains unclear. Thus far, low levels of p53 have only been described to induce temporary CCA. In case of irreversible DNA damage, p53 plays a prominent role in DDR-mediated apoptosis via induction and inhibition of pro-apoptotic and anti-apoptotic proteins, respectively (reviewed by ref. (Khoo et al. 2014)). Pro-apoptotic proteins like phorbol-12-myristate-13-acetate-induced protein 1 (PMAIP1; also known as NOXA) and p53-upregulated modulator of apoptosis (PUMA) bind and inhibit multiple mitochondrial anti-apoptotic BCL family members (Chen et al. 2005). Pro-apoptotic proteins that are directly upregulated by p53 also include BAX and p53-inducible protein 3 (PIG3) (Samuels-Lev et al. 2001).

The exact mechanisms of p53 and its impact on the pro/anti-apoptotic balance in senescence remain unclear. Strikingly, senescent IMR90 cells show upregulation of pro-apoptotic PUMA and BIM and reduced anti-apoptotic BCL-2, but this does not induce these cells to actually undergo apoptosis (Baar et al. 2017). This evasion of apoptosis could be explained by secondary factors that influence the function of p53, thereby favoring senescence over apoptosis. Such factors include the Forkhead box protein O4 (FOXO4) that binds and retains p53 to persistent nuclear foci containing DDR proteins termed ‘DNA segments with chromatin alterations reinforcing senescence’ (DNA-SCARS) that associate with PML (promyelocytic leukemia protein) nuclear bodies (Rodier et al. 2011; Baar et al. 2017). The association between DNA-SCARS en PML nuclear bodies, where many repair and chromatin-modifying proteins localize, promotes senescence-associated gene expression (Rodier et al. 2011). An anti-apoptotic role has also been assigned to CCN2 in various organs and tissues, including mesangial cells of human kidneys (Wahab et al. 2007).

## Evidence for senescence in the kidney from observational studies

It is a well-established fact that senescent cell numbers are increased during physiological renal aging as well as in response to renal injury. Tables 2 and 3 lists available reports to date, regarding quantification of senescent cell numbers in the human kidney during renal physiological aging and disease, and their relation with glomerulosclerosis and/or interstitial injury. To what extent senescence might be an epiphenomenon, or causally linked to clinically relevant parameters such as renal interstitial and/or glomerular disease and renal functionality, remains controversial and is further discussed below.

**Table 2:** Observations regarding quantification of senescent cell numbers in renal aging

Study	Species (comparison or range in age)	Senescence marker	Sublocalization	Association with aging
Aging				
(Krishnamurthy et al. 2004)	Mouse and rat (3 vs 28 months)	SA-β-Gal, p16, p19	Cortical tubules	Nephritis
(Chkhotua et al. 2003)	Human (21–80 years)	p16, p27	Cortical tubules and Interstitium	Nephron atrophy
(Melk et al. 2004)	Human (8 weeks-88 years)	p16, p53, TGFβ1, p14	Glomeruli, tubules, arteries	Glomerulosclerosis, interstitial fibrosis and tubular atrophy
(Ding et al. 2001)	Rat (3 vs12 vs 24 months)	SA-β-gal, TGF-β1, p21	Tubulointerstitium	Interstitial fibrosis and tubular atrophy
(Melk et al. 2003)	Rat (9–24 months)	p16, SA-β-gal	Glomeruli, tubulointerstitium	Interstitial fibrosis
(Sis et al. 2007)	Human (mean age 36.4 years)	p16	Glomeruli, tubulointerstitium, arteries	None
Aging in (a model of) renal transplantation				
(Melk et al. 2009)	Mouse (3 vs 18 months)	p16, Ki-67	Glomeruli, tubulointerstitium	Tubular atrophy, reduced tubular proliferation
(Clements et al. 2013)	Mouse 8–10 vs 46–49 weeks)	SA-β-Gal, p53, p21	Tubules	Mortality and kidney function, interstitial fibrosis, inflammation
(Chkhotua et al. 2003)	Human (19–60 years)	p16, p27	Glomeruli, tubulointerstitium	None

**Table 3:** Observations regarding quantification of senescent cell numbers in renal disease

Study	Disease or context	Species	Senescent marker	Sublocalization	Association
Renal disease					
(Westhoff et al. 2008)	Hypertension	Human	p16	Glomeruli, tubulointerstitium, arteries	Tubular atrophy, interstitial fibrosis, glomerulosclerosis and vascular damage
(Liu et al. 2012b)	IgA nephropathy	Human	SA-β-Gal, p16, p21	Tubules	Blood pressure, disease progression, glomerulosclerosis, tubular atrophy, interstitial fibrosis, inflammatory cell infiltration, matrix accumulation,
(Verzola et al. 2008)	Diabetic nephropathy	Human	SA-β-Gal, p16	Glomeruli, tubules	BMI, blood glucose, proteinuria, LDL cholesterol, HbA1c, glomerular ischemic lesions, tubular atrophy
(Kitada et al. 2014)	Diabetic nephropathy (STZ)	Mouse	SA-β-Gal, p21	Glomeruli, tubules	None
(Sis et al. 2007)	Glomerular disease (MN, FSGS and MCD)	Human	p16	Glomeruli, tubulointerstitium	Proteinuria, age, tubular atrophy and interstitial fibrosis, interstitial inflammation
(Park et al. 2007)	ADPKD	Human and rat	p21 (decreased)	Not assessed	None
(Lu et al. 2016)	Nephronophtisis	Mouse	SA-β-Gal, p16	Tubules	None
(Quimby et al. 2013)	Chronic kidney disease	Cat	SA-β-Gal	Tubules	Telomere shortening
Therapy induced					
(Zhou et al. 2004)	Cisplatin	Rat	p21, p27	Tubules	DNA repair
(Melk et al. 2005)	Delayed graft function and diseased native kidneys	Human	p16	Glomeruli, tubulointerstitium, arteries	Tubular atrophy and interstitial fibrosis (delayed graft function)
(Chkhotua et al. 2003)	CAN	Human	p16 and p27	Glomeruli, tubulointerstitium	Severity of CAN
G2-arrest					
(Bonventre 2014b)	Multiple tubular injury	Mouse	TGF-β1	Not assessed	Increased creatinine, glomerulosclerosis, tubular atrophy, interstitial fibrosis, myofibroblast proliferation, vascular rarefaction

Abbreviations: STZ = streptozotocin; MN = membranous nephropathy, FSGS = focal segmental glomerulosclerosis, MCD = minimal change disease; ADPKD = autosomal dominant polycystic kidney disease; CAN = chronic allograft nephropathy

### Senescence in renal aging

To date no published data are available that specifically address G2 arrest in aging kidneys. However, varying combinations of senescence markers, such as p16 and SA-β-Gal, have consistently been found to be increased in aged kidneys (Table 2)*.* Thus, increased numbers of senescent cells were found in association with glomerulosclerosis and tubulointerstitial changes (Ding et al. 2001; Chkhotua et al. 2003; Melk et al. 2003; Melk et al. 2004). Additionally, in a murine model of allograft nephropathy, old donor kidneys displayed increased p16 levels, a reduced proliferation of tubular epithelial cells after transplantation, and increased susceptibility to transplantation related stress compared to kidneys from young donors (Melk et al. 2009). Also, old mice display a relatively higher increase of SA-β-Gal, p53 and p21, compared to young mice upon IRI (Clements et al. 2013). *()* Together, these observations suggest an age-dependent increase in the susceptibility of the kidney to induction of senescence and concomitantly reduced regenerative capacity.

### Senescence in renal disease

In response to injury, renal senescent cell numbers, indicated by varying combinations of markers mainly consisting of SA-β-Gal, p16 and p21 expression, are increased in various experimental animal models and human renal diseases (Table 3). For example, an increase of senescence markers was observed upon renal injury in rodent models including DOCA-salt-induced hypertension, streptozotocin (STZ)-induced diabetic nephropathy (DN), and cisplatin-induced nephrotoxicity (Zhou et al. 2004; Westhoff et al. 2008; Kitada et al. 2014). Increased expression of senescence markers has also been found in diseased human and mouse kidneys in hypertension, DN, CKD, delayed graft function (DGF) after kidney transplantation, and in various glomerular diseases, including membranous nephropathy, minimal change disease, IgA nephropathy (IgAN) and focal segmental glomerulosclerosis (FSGS)) (Melk et al. 2005; Sis et al. 2007; Westhoff et al. 2008; Verzola et al. 2008; Liu et al. 2012a; Quimby et al. 2013). Importantly, in hypertension, DN, MG, IgAN, FSGS and also in DGF after renal transplantation, senescent cell accumulation correlated with renal histopathological changes (glomerulosclerosis, tubular atrophy, and interstitial fibrosis), with renal function, and/or with proteinuria, while in IgAN, tubular expression of p16, p21 and SA-beta-gal also correlated with blood pressure(Liu et al. 2012b). In DN, tubular SA-β-Gal expression correlates with body mass index (BMI) and blood glucose, and tubular p16 is associated with BMI, LDL cholesterol and HbA1c (Verzola et al. 2008). Furthermore, the increment of renal senescent cells was associated with disease progression in IgAN (Liu et al. 2012b).

In line with these observations, therapeutic intervention in rodents reduced the accumulation of senescent cells induced by DOCA-salt, STZ and cisplatin respectively (Zhou et al. 2004; Westhoff et al. 2008; Kitada et al. 2014)*.* Remarkably, only one study failed to show a relation between senescent cell accumulation and disease progression, where in kidney biopsies from diabetic patients with proteinuria, both p16 and SA-β-Gal were strongly upregulated in an early phase, but did not further increase during disease progression (Verzola et al. 2008).

Taken together, these data indicate that cellular senescence is associated with detrimental effects contributing to histopathological and functional deterioration and that it can be caused by various distinct disease-associated triggers. Moreover, it appears that in various diseases of the kidney senescence might be prevented or overcome by the application of appropriate therapeutic strategies. Thus, disease- and therapy-induced damage might induce renal senescence and contribute to histopathological and functional changes in the kidney and even play a deleterious role in disease progression. These detrimental effects of senescence could be explained by (i) reduced organ function via SASP effects, (ii) persistence of functionally incompetent cells, and (iii) impairment of the proliferation required to replace damaged cells.

## Type and localization of renal senescent cells

In the aging or injured kidney, senescent cells have been found in both the medulla and cortex, and include tubular, glomerular, interstitial and vascular cells. Senescent cells are predominantly found in the cortex and represent mainly proximal tubular cells (PTC). This holds true especially G2-arrested senescent cells (Yang et al. 2010; Bonventre 2014a). Markers of senescence are also found in glomeruli of diseased kidneys, including DN, membranous nephropathy, FSGS, minimal change disease and glomerulonephritis (Melk et al. 2005; Sis et al. 2007; Verzola et al. 2008). The affected cell types in these conditions are podocytes, mesangial and/or endothelial cells, and parietal epithelia cells. Interstitial and vascular cells are among the cell types undergoing senescence in hypertension and glomerular disease (Sis et al. 2007; Westhoff et al. 2008). Thus, the type and localization of senescent cells seems to be dependent on the specific stressors involved and on the exact location of injury. In the context of aging, senescence might be induced mainly in tubular cells due to increased oxidative and cellular stress (Melk et al. 2004). Although tubular epithelium also seems to account for the majority of senescent cells in renal disease, other cell types may be affected as well, corresponding with the location of injury. For instance, kidneys with glomerular disease typically display increased expression of p16 in glomerular cell nuclei (Sis et al. 2007).

## Eliminating senescent cells in renal injury

Possible benefits of intervention in the process of senescence have been explored through transgenic depletion and pharmaceutical inhibition or elimination of senescent cells (i.e. senotherapy). Table 4 summarizes the data published to date regarding the depletion or inhibition of formation of senescent kidney cells in experimental kidney injury. Many mouse studies have shown already that depletion/inhibition of senescent cells via genetic modification or pharmaceutical inhibition reduces renal injury, while senotherapeutics have been studied less extensively in the kidney and are discussed in the next section. In the context of aging, senescent cell depletion through the INK-ATTAC transgene that removes p16(Ink4a)-positive senescent cells upon drug treatment, leads to attenuated glomerulosclerosis and lower blood urea nitrogen levels later in life (Baker et al. 2016). In contrast, knockdown of p16 increased renal fibrosis following unilateral ureteral obstruction (UUO), indicating a beneficial effect of senescence on tissue remodeling upon acute kidney injury (Wolstein et al. 2010). Similarly, deletion of p21 and autophagy protein 5 (ATG5, which is critically involved in tubular epithelial senescence) aggravated ischemia-reperfusion injury (IRI), leading to increased renal damage and cell death, impaired renal recovery and higher mortality (Megyesi et al. 2001; Baisantry et al. 2016). On the other hand, inactivation of p16 or ATG5 resulted in reduction of interstitial fibrosis and nephron atrophy later after IRI, indicating a protective long-term effect of inhibition of senescence on the development of fibrosis (Lee et al. 2012; Braun et al. 2012; Baisantry et al. 2016). Furthermore, pharmaceutical p53 and JNK inhibition led to reduced numbers of G2-arrested cells together with less fibrosis in a model of severe bilateral IRI (Yang et al. 2010).

**Table 4:** Outcome of intervention of senescence in renal aging and after kidney injury in mice

Study	Model of kidney injury	Method of senescence intervention	Acute and long-term outcome (days after kidney injury)	Effect of senescence
(Baker et al. 2016)	Natural aging (1-year old)	p16-KO	Attenuated glomerulosclerosis	Detrimental: contributing to renal aging
(Wolstein et al. 2010)	UUO	p16-KO	Acute (10d): increased renal fibrosis	Beneficial: part of anti-fibrotic mechanism
(Megyesi et al. 2001)	IRI	p21-KO	Acute (<7d): impaired renal recovery, higher renal damage, higher mortality	Beneficial: responsible for recovery after acute ischemic renal failure
(Baisantry et al. 2016)	IRI	ATG5-KO	Acute (3d): increased renal damage, increased cell death Long-term (30d): attenuated interstitial fibrosis, better kidney function	Beneficial: responsible for recovery after acute ischemic renal failure Detrimental: promoting renal fibrosis
(Lee et al. 2012)	IRI	INK4a-KO	Long term: improved kidney regeneration (14d), decreased capillary rarefaction (1-28d)	Detrimental: promoting renal fibrosis
(Braun et al. 2012)	Kidney transplantation	p16-KO	Long term (21d): reduced interstitial fibrosis, reduced nephron atrophy	Detrimental: contributing to adverse long-term allograft outcomes
(Hochegger et al. 2007)	IRI	p53 inhibition via pifithrin-α	Acute (<48 h of reperfusion): reduced serum creatinine, reduced tubular necrosis score	Detrimental: contributing to acute renal failure after ischemia
(Yang et al. 2010)	IRI, AAN, UUO	p53 inhibition via pifithrin-α	Reduced fibrosis	Detrimental: promoting renal fibrosis

Abbreviations: IRI = ischemia reperfusion injury; UUO = unilateral ureteral obstruction; AAN = acute aristolochic acid toxic nephropathy; KO = genetic knock-out

**Fig. 3:**
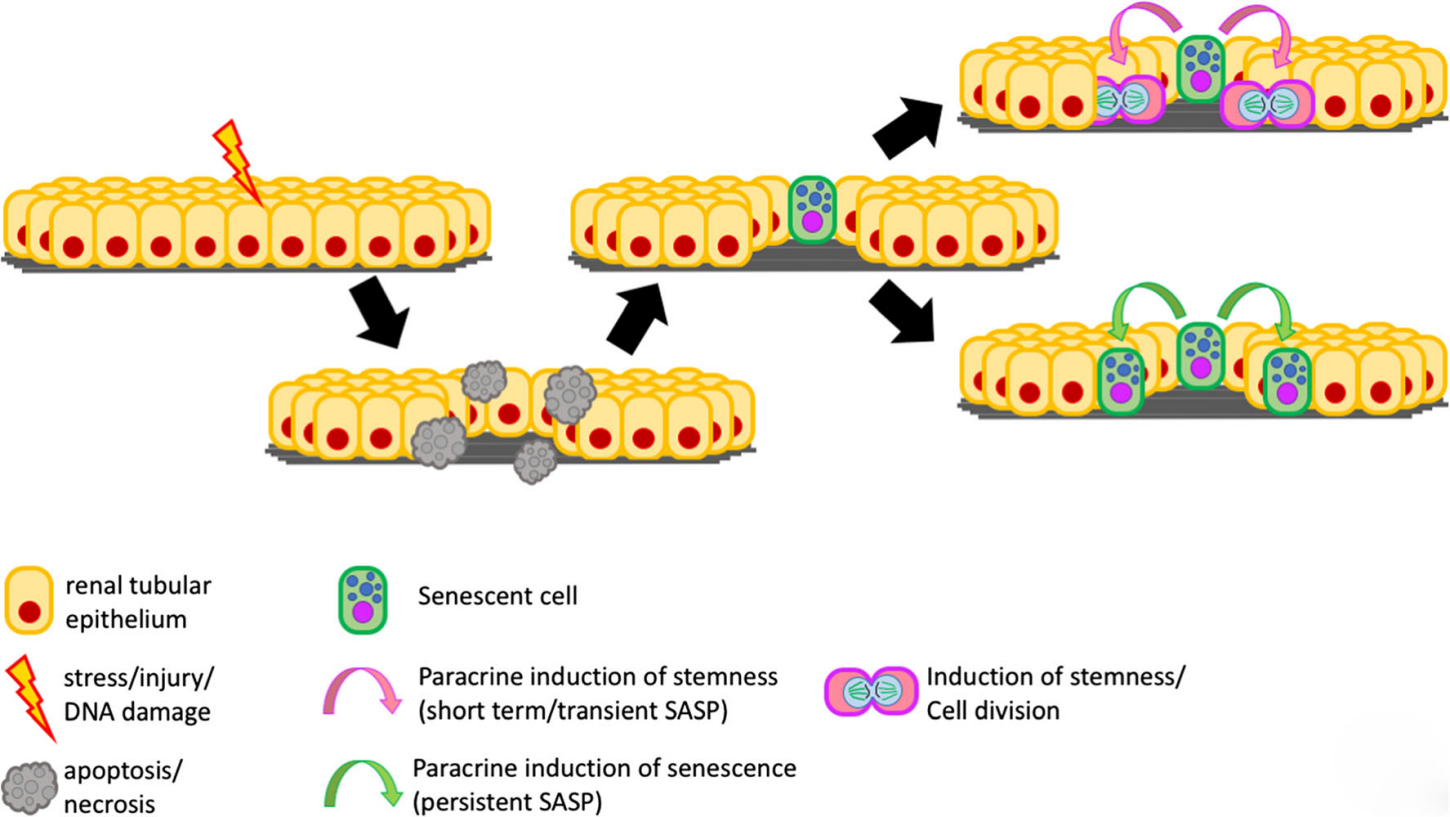
Paracrine effects of senescent cells in early and late phases of tissue injury

In summary, the fact that senescent-cell depletion induces a maladaptive, fibrotic repair response in UUO-related obstructive injury and in in the acute phase after IRI, while it leads to less apoptosis and enhanced regenerative proliferation in the chronic phase after IRI (O'Sullivan et al. 2017) points to a beneficial effect of cellular senescence in the early phase of acute kidney injury, where it might support regeneration while, in contrast, prolonged senescence during later stages appears to have detrimental effects in more chronic renal injury, a feature demonstrated in Fig. 3. Of note, similar paradoxical effects have been attributed to cellular senescence also in other conditions, including tumor biology and liver regeneration, where SASP can mediate paracrine effects of senescent cells, inducing either stemness or senescence in neighboring cells, depending on short or long duration, respectively (Ritschka et al. 2017)*.* Obviously, this time-dependent effect on outcome will be of key importance for translational opportunities. For example, in kidney transplantation, acute as well as chronic factors may drive accumulation of senescent cells. These include tacrolimus nephrotoxicity which, in rodents, involves the production of ROS and subsequent DDR, and possibly also reduction of physiological clearance of senescent cells as a result of immunosuppressive therapies (Khanna and Pieper 2007).

The interpretation and applicability of experimental studies with reference to human kidney aging and disease is complex. Renal disease models are mostly performed in young rodents and of relatively short duration. They thereby reflect acute, rather than chronic kidney injury and may be most relevant to studying the role of senescence in acute, regenerative responses to injury (Le Clef et al. 2016). Furthermore, it should be kept in mind that most rodent experimental models of renal disease are performed in relatively young animals, potentially affecting their relevance to the aging kidney. As for understanding the role of cellular senescence in progression from acute to chronic kidney injury, and in CKD itself. The chronic IRI and multiple hit models with their cumulative, diverse stresses, and also the observations on senescent cell depletion during normal and accelerated aging will be most relevant (Bonventre 2014a; Le Clef et al. 2016; Sturmlechner et al. 2017).

## Pharmaceutical senotherapies

Clearance of senescent cells through transgenic depletion has revealed promising beneficial effects on kidney homeostasis during aging and upon damage. For translational purposes, development of non-transgenic intervention is now of major interest and treatment with several different compounds targeting a variety of senescence-associated pathways are under study. These so-called senotherapies can be broadly divided according to three different approaches (Childs et al. 2015):

## Prevention of senescence

The first approach is to prevent senescence by limiting triggers inducing senescence through lifestyle and anti-aging drugs (e.g. caloric restriction, antioxidant agents, etc.), and by inhibiting pro-inflammatory pathways. The role of cellular senescence in the renal effects of successful lifestyle interventions linked with extended (healthy) lifespan, such as a healthy diet, exercise and avoidance of smoking, is not clear. However, such a role would be compatible with the known efficacy of caloric restriction in rats, where it extended healthy lifespan and also reduced oxidative DNA damage, pro-inflammatory factors, senescence and fibrosis in the kidney (Heydari et al. 2007; Ning et al. 2013; Xu et al. 2015). The life extending effect of caloric restriction in rodents is mediated through the MAPK and mTOR growth promoting pathways, which are linked to the SASP (Inoki et al. 2012). Antioxidants might be beneficial by reducing ROS-mediated DNA damage and thereby preventing DDR-induced senescence (Holmström and Finkel 2014). Mitochondria-targeted therapy with SS-31 (also known as Elamipretide) is thought to stabilize cardiolipin on the inner mitochondrial membrane, thereby limiting mitochondrial dysfunction (Kloner et al. 2015). Administration of this peptide reduces senescence of renal parietal epithelial cells (PECs) in aged mice, accompanied by attenuated glomerulosclerosis in treated mice compared to controls (Sweetwyne et al. 2017). The authors link these results to the detrimental effect of mitochondrial dysfunction, indicated by the observations that PECs of treated mice display reduced mitochondrial damage (also observed in podocytes), reduced upregulation of the ROS-generating enzyme Nox4, and reduced senescence compared to controls. Moreover, 8 weeks of treatment with Elamipretide results in increased PEC density and attenuated PEC activation, but also led to reduced podocyte injury, and increased glomerular endothelial capillary integrity. Metformin also reduces the production of ROS (Algire et al. 2012).

## Elimination of senescent cells

The second approach is to aim at the removal of senescent cells. So called ‘senolytics’ interfere with anti-apoptotic and pro-survival signaling, thereby eliminating senescent cells (Zhu et al. 2015). Senescent cells have much in common with cancer cells, including similarities in metabolic activity, the DDR and activation of pro-survival pathways or inhibition of pro-apoptotic pathways (Ghosal and Chen 2013; Dörr et al. 2013). Therefore, similar strategies used for mediating apoptosis in cancer cells, are explored for removal of senescent cells. In other words, antitumor drugs are investigated for their potential as senolytic agents. ABT-263 (also known as navitoclax) is one of the most widely studied senolytic agents. It is regarded as a pan-BCL inhibitor, as it is known to cause apoptosis in various cell types by inhibiting the anti-apoptotic BCL family members, including BCL-2, BCL-XL and BCL-W (Chang et al. 2016; Yosef et al. 2016). However, navitoclax has not yet been tested for its impact on viability, phenotype, and function of kidney cells in vitro or in vivo. Other agents studied for targeting senescent cells *(although also not in the kidney)* include the cancer drugs quercetin and dasatinib (Zhu et al. 2015). These anti-tumor agents are known for inhibiting a broad spectrum of protein kinases and tyrosine kinases(O'Hare et al. 2005; Russo et al. 2012). In vitro, quercetin and dasatinib reduce expression of the anti-apoptotic regulator PAI-2 and induce apoptosis in senescent primary human pre-adipocytes and HUVECs, respectively (Zhu et al. 2015). In vivo, combined administration of quercetin and dasatinib leads to reduced markers of senescence (SA-β-Gal and p16) in fat and liver tissue from old mice, and to functional improvement. However, the effect of these drugs has been reported to be non-specific as it remains unclear in how far they are due to senescent cell depletion, or secondary to intervention in a multitude of unrelated pathways (Chang et al. 2016). More recently, the selective BCL-XL inhibitors A1331852 and A1155463, were identified as potential senolytics, inducing senescence-specific apoptosis in human umbilical vein endothelial cells (HUVECs) and human lung fibroblast (IMR90) (Zhu et al. 2017). As selective BCL-XL inhibitors, they may have less hematological toxicity than navitoclax.

Elimination of senescent cells may also be induced by Fisetin, a naturally occurring flavone that causes senescence-specific apoptosis in HUVECs, and by the natural product piperlongumine which caused apoptosis of senescent human WI-38 fibroblasts, no matter whether senescence was induced by either ionizing radiation, or replicative exhaustion, or ectopic expression of the Ras oncogene (Zhu et al. 2016; Wang et al. 2016). The precise mechanism of action by which fisetin and piperlongumine induce apoptosis in senescent cells still remains unclear.

Another possible target for interfering in the apoptotic balance is CCN2 which is part of the SASP. In human mesangial cells, CCN2 stabilizes the anti-apoptotic protein Bcl-2, by activation of MAPK phosphatase-1 (MKP-1), resulting in reduced apoptosis and cell survival (Wahab et al. 2007). In addition, CCN2 plays a key role in the pathogenesis of kidney disease and can activate profibrotic pathways. Theoretically, targeting senescence-induced CCN2 might thus have beneficial effects at multiple levels, including triggering apoptosis of senescent cells. Indeed, genetic silencing of MKP-1 using siRNA or antisense oligonucleotides was able to induce cell apoptosis in mesangial cells treated with CCN2 (Wahab et al. 2007). However, direct evidence for CCN2-induced upregulation of anti-apoptotic proteins in the context of senescence is lacking. Additionally, the tumor suppressor p53, a known target for cancer treatment, can also be targeted for halting senescence. The FOXO4-D-Retro-Inverso(DRI) peptide (also known as Proxofim) selectively induces targeted apoptosis of senescent cells (TASC) by competing with normal anti-apoptotic FOXO4-p53 binding (Baar et al. 2017). Similar to trans-genetic elimination of senescent cells discussed above, administration of FOXO4DRI cell-penetrating peptide reduced tubular senescent cell numbers, preserving and even restoring renal function in both rapidly aging trichothiodystrophy (TTD) mice and naturally aging wild type mice. As discussed above, these promising data should be seen in the context that several possible adverse effects of eliminating senescent cells have been pointed out, including impaired cutaneous wound healing and increased fibrosis upon liver damage, underscoring the critical positive contribution of naturally occurring cellular senescence to (acute) regenerative processes (Krizhanovsky et al. 2008; Demaria et al. 2014). Another, maybe largely theoretical, consideration might be that application of senolytics in advanced disease states with high numbers of accumulated senescent cells, might lead to a cell lysis syndrome due to sudden elimination of massive numbers of senescent cells. In most known conditions, however, this seems unlikely as senescent cells only seem to make up for a small percentage of total cells.

## Modulation of the SASP

SASP modulating drugs target pro-inflammatory signaling pathways such as NF-κB, JNK or p38 MAPK. SP600125 is an inhibitor of c-Jun N-terminal kinase (JNK), a member of the growth promoting pathway MAPK playing an essential role in inflammatory responses, including the SASP (Bennett et al. 2001). In the kidney, G2-arrested PTCs activate JNK-signaling, thereby upregulating profibrotic cytokines like CCN2 and TGF-β As discussed above (Yang et al. 2010), IRI after treatment with a SP600125 (a pan-JNK inhibitor) leads to lower numbers of senescent cells in G2-phase and reduces fibrosis (Yang et al. 2010). The pro-inflammatory pathways of the SASP can also be targeted by the mTOR inhibitor rapamycin or the AMPK activator metformin, leading to prevention of senescence (Iglesias-Bartolome et al. 2012; Noren Hooten et al. 2016). Although SASP modulation is expected to limit detrimental paracrine effects of prolonged presence of senescent cells, SASP modulation could also lead to harmful side-effects. First, SASP factors are not senescence-specific but are upregulated in a broad spectrum of different pathways, and intervention could thus interfere with vital processes. Secondly, SASP modulation could impede immune surveillance, and hamper elimination of senescent cells.

## Stimulating senescence

Strikingly, certain triggers inducing senescence ultimately limit rather than stimulate fibrosis in various organs and conditions. For instance, the matricellular protein CCN1 (also known as CYR61) induces senescence of fibroblasts in cutaneous wound healing and of liver myofibroblasts in hepatic injury, and its expression is linked to reduced fibrosis (Jun and Lau 2010; Kim et al. 2013). Therefore, CCN1-induced senescence might be used as therapy to limit fibrosis. Although its role in the kidney remains unclear, CCN1 expression is also elevated in several senescence-associated human pathologies beyond the liver, including atherosclerosis, which suggests a possible role in age-related diseases (Littlewood and Bennett 2007). On the other hand, the expression of CCN2, another, closely related, member of the CCN protein family, is associated with fibrosis of different organs, including the liver (Krizhanovsky et al. 2008). This indicates that the dual role of senescence on fibrosis is dependent on varying triggers.

## Targeting strategies

Currently available senolytic drugs have several limitations, the major challenge being to target the right cells at the right time. The clinical applicability of systemically administered senolytic drugs is impeded by dose limiting side-effects on other organs. For instance, navitoclax caused dose-dependent thrombocytopenia, due to inhibition of BCL-X in platelets resulting in platelet apoptosis (Mason et al. 2007; Zhang et al. 2007; Wilson et al. 2010). Theoretically, such limitations of senolytic drugs may be overcome by targeted delivery to specific organs and cell types or by specific targeting of senescent cells.

Targeted accumulation of senolytic agents in the kidney might be achieved using nanomedicines (i.e. nanoparticulate carriers) like conjugates and liposomes. Delivery of therapeutic of such functionalized compounds should enable high enough drug concentrations where needed, while sharply limiting systemic drug exposure and thereby side effects. The delivery of an effective senolytic drug dose inside targeted cells would be accomplished by internalization of the functionalized compound triggered by binding to the cell surface receptors specifically expressed by certain cell types in the kidney (Falke et al. 2015). There is a variety of compounds that can carry therapeutic agents, including glucosamine conjugates, poly vinylpyrrolidone (PVP)-derivatives, lysozyme conjugates and other low molecular weight protein (LMWP)-carriers, and also the targeting peptide (KKEEE)3 K (Franssen et al. 1993; Kamada et al. 2003; Lin et al. 2013; Falke et al. 2015; Wischnjow et al. 2016). The kidney is eminently qualified for such targeting strategies, as kidney cells possess several relatively specific cell surface receptors, including LDL receptor-related protein 2 (commonly known as megalin/cubulin), integrin α8, E-selectin, podocyte-specific antigen and folate receptor 1α (Falke et al. 2015).

Targeting of PTCs, which display features of senescence with aging and pathology, may be the most promising approach, as these cells are highly active in accumulating compounds from the filtered urine via their receptors in the luminal brush-border (Christensen et al. 2012). Among these, megalin and folate receptor 1α have been successfully employed for targeting the proximal tubular epithelium (Prakash et al. 2008; Shillingford et al. 2012). Another promising approach to target PTCs is the therapeutic use of small interfering RNA (siRNA). In mouse models of ischemic and cisplatin-induced acute kidney injury, intravenous administration of siRNA against p53 reduces cellular p53 and attenuates p53-mediated apoptosis (Molitoris et al. 2009). The validity of this approach is currently being addressed in a phase 3 clinical trial testing p53 siRNA for preservation of kidney function after major cardiothoracic surgery.

Targeting other renal cell types (i.e. mesangial cells, endothelial cells and podocytes) is theoretically possible through targeting of different surface receptors, using nanoparticles, or with liposomes(Tuffin et al. 2005; Choi et al. 2011; Kamaly et al. 2016). For instance, mesangial cells, that predominantly become senescent as a consequence of hypertension and diabetic nephropathy, have been targeted through integrin α8 and Thy 1.1 using liposomes in rodents(Scindia et al. 2008; Suana et al. 2011).

## Summary and future

In the kidney, both G1- and G2-arrested senescent cells accumulate with advancing age and renal disease in various areas, particularly in cortical proximal tubular cells. In aging mice, genetic clearance of senescent cells leads to better preservation of kidney function and morphology. In diseased kidneys, there is a time-dependent effect of senescence on the development of fibrosis, with early beneficial effects and detrimental long-term consequences. Studies examining therapeutic options for depletion of senescent cells in humans are complicated because of dose limiting side effects on other organs. Therefore, specific targeting of senescent cells in the kidney might be essential.

Further research is also needed to understand in how far accumulation of renal senescent cells in renal aging and disease (i) is due to increased production or reduced clearance via immune surveillance, (ii) is a direct cause or a consequence of progressive organ injury and organismal aging and (iii) if elimination of these cells (at the right time) improves kidney function and histopathological changes. Novel therapeutic approaches for elimination of senescent cells in vivo, including targeting strategies to overcome dose limiting side effects on other organs, will be important to find answers to these questions.
